# Hyperaemia during dynamic handgrip exercise is preserved in healthy young subjects after recovery from COVID‐19

**DOI:** 10.1113/EP091656

**Published:** 2024-03-09

**Authors:** Eliza Prodel, Roberto Souza, Beatriz Divino, Helena N. M. Rocha, Natalia G. Rocha, Antonio C. L. Nobrega

**Affiliations:** ^1^ Laboratory of Exercise Science, Department of Physiology and Pharmacology Fluminense Federal University Niterói Brazil; ^2^ Laboratory of Integrative Cardiometabology, Department of Physiology and Pharmacology Fluminense Federal University Niterói Brazil

**Keywords:** blood flow, COVID‐19, dynamic exercise, SARS‐COV‐2

## Abstract

We sought to investigate possible impaired hyperaemia during dynamic handgrip exercise (HGE) in young healthy individuals who had recovered from COVID‐19. We tested the vascular function in individuals recovered from COVID‐19 using a nitric oxide donor (i.e., sodium nitroprusside; SNP), which could revert a possible impaired endothelial function during HGE. Further, we tested whether individuals who recovered from COVID‐19 would present exaggerated brachial vascular resistance under an adrenergic agonist (i.e., phenylephrine; PHE) stimuli during HGE. Participants were distributed into two groups: healthy controls (Control; men: *n* = 6, 30 ± 3 years, 26 ± 1 kg/m^2^; and women: *n* = 5, 25 ± 1 years, 25 ± 1 kg/m^2^) and subjects recovered from COVID‐19 (post‐COVID; men: *n* = 6, 29 ± 3 years, 25 ± 1 kg/m^2^; and women: *n* = 10, 32 ± 4 years, 22 ± 1 kg/m^2^). Participants in the post‐COVID group tested positive (RT‐PCR) 12–14 weeks before the protocol. Heart rate (HR), brachial blood pressure (BP), brachial blood flow (BBF) and vascular conductance (BVC) at rest were not different between groups. The HGE increased HR (Control: Δ9 ± 0.4 bpm; and post‐COVID: Δ11 ± 0.4 bpm) and BP (Control: Δ6 ± 1 mmHg; and post‐COVID: Δ12 ± 0.6 mmHg) in both groups. Likewise, BBF (Control: Δ632 ± 38 ml/min; and post‐COVID: Δ620 ± 27 ml/min) and BVC (Control: Δ6.6 ± 0.4 ml/min/mmHg; and post‐COVID: Δ6.1 ± 0.3 ml/min/mmHg) increased during HGE. SNP did not change HGE‐induced hyperaemia but did decrease BP, which induced a reflex‐related increase in HR. PHE infusion also did not change the HGE‐induced hyperaemia but raised BP and reduced HR. In conclusion, exercise‐induced hyperaemia is preserved in healthy young subjects 12–14 weeks after recovery from COVID‐19 infection.

## Abstract (highlights)

- **What is the central question of this study?**
  How does vascular function compare during dynamic handgrip exercise in young healthy adult males and females who recovered from COVID‐19?
- **What is the main finding and its importance?**
  No differences were observed in exercise hyperaemia in healthy young adults, males and females, who recovered from COVID‐19 compared to the healthy control group. Vascular function seems to be preserved in individuals 12–14 weeks after COVID‐19 during dynamic handgrip exercise in young healthy adults, males and females.

## 1 INTRODUCTION

Pathophysiological alterations of the respiratory syndrome coronavirus 2 (SARS‐Cov‐2) disease (COVID‐19) have resulted in long‐lasting sequelae in the cardiovascular system (Davis et al., 2021). Acute SARS‐Cov‐2 infection of endothelial cells induces endothelial dysfunction, thereby explaining a possible reduced NO‐mediated vasodilatation. However, whether endothelial function is preserved weeks after recovery from COVID‐19 acute infection is not known (Xu et al., 2023).

Young healthy adults, 4 weeks after recovery from mild‐symptom COVID‐19, showed exaggerated blood pressure (BP) and heart rate (HR) response and decreased exercise‐induced hyperaemia during dynamic handgrip exercise (HGE) (Stute et al., 2022). Additionally, flow‐mediated dilatation, a marker of vascular endothelial function, was impaired 4 weeks after recovery from COVID‐19 (Davis et al., 2021; Yong, 2021). However, the time course for the recovery of those vascular impairments and possible underlying physiological mechanisms are unknown. Additional to endothelial dysfunction, exaggerated vascular transduction of sympathetic stimulation via α‐adrenergic receptors could also explain impaired exercise‐induced hyperaemia during dynamic exercise in young men and women after recovery from COVID‐19 (Stute et al., 2022).

Locally released substances during skeletal muscle contraction overcome α‐adrenergic vasoconstriction allowing an increase of blood flow to match exercise metabolic demand (Remensnyder et al., 1962; Saltin & Mortensen, 2012). The mechanisms which culminate in vasodilatation during skeletal muscle contraction are not precisely known; however, endothelial nitric oxide (NO) release plays an important role (Clifford & Hellsten, 2004; Remensnyder et al., 1962). Hence, a decrease in vasodilatation and blood flow to exercising muscles during dynamic exercise could explain the exercise intolerance and fatigue observed post‐COVID‐19 infection (Haffke et al., 2022).

In young healthy men and women who recovered from COVID‐19 compared to matched controls, we sought to investigate (1) possible impaired exercise‐induced hyperaemia, (2) whether a NO donor (i.e., sodium nitroprusside; SNP) would revert this possible impaired exercise‐induced hyperaemia, and (3) exaggerated brachial vascular resistance under adrenergic agonist (i.e., phenylephrine; PHE) stimuli during dynamic HGE.

## 2 METHODS

### 2.1 Participants

All experimental procedures were approved by the Fluminense Federal University Antonio Pedro University Hospital ethics committee (CAAE: 50132121.3.0000.5243; approval: 5.002.744) and conducted according to the *Declaration of Helsinki*, aside from registration in a clinical database (World Medical Association, 2013) from April to October 2022. After receiving detailed verbal and written information about the protocol, each participant signed a consent form. Twenty‐seven healthy young men and women volunteered to participate in the protocol. Those participants were recruited by convenience and were distributed into two groups: healthy controls (Control; men: *n* = 6, 30 ± 3years, 26 ± 1 kg/m^2^; and women: *n* = 5, 25 ± 1 years, 25 ± 1 kg/m^2^) and subjects recovered from COVID‐19 (post‐COVID; men: *n* = 6, 29 ± 3 years, 25 ± 1 kg/m^2^; and women: *n* = 10, 32 ± 4 years, 22 ± 1 kg/m^2^). Participants in the post‐COVID group tested positive (RT‐PCR) 12–14 weeks before the protocol. During the acute infection, participants reported mild to moderate flu‐like symptoms; none of them needed hospitalization. The control group did not present nor had contact with people who presented flu‐like symptoms since the outbreak of the pandemic. All participants were vaccinated with the booster dose, and to participate in the protocol, they had to report no symptoms of cardiovascular, neurological, respiratory, renal, metabolic, or hepatic diseases, or signs and symptoms of long COVID. Women were tested during the early follicular phase of the menstrual cycle, or during the low‐hormone phase of oral contraceptive use, to minimize the effects of reproductive hormone variation across the menstrual cycle, and had not been breastfeeding for at least 2 years. Participants were not using prescribed or over‐the‐counter medication including primary dysmenorrhoea therapy. Participants were asked to abstain from caffeinated and alcoholic beverages and exercise for 24 h before the experimental session. The experimental protocol was always conducted during the morning. The room was maintained at a controlled temperature, between 22 and 24°C.

### 2.2 Experimental measurements

HR (bpm) was continuously monitored using a standard lead II electrocardiogram. Systolic and diastolic blood pressure (SBP and DBP, mmHg) were measured beat‐to‐beat by photoplethysmography from the middle finger of the left hand. Those signals were sampled at 1000 Hz and integrated for offline analysis (PowerLab, LabChart; ADInstruments, Bella Vista, Australia). Brachial BP was measured on the left arm using the auscultatory method (TANGO M2, SunTech Medical, Morrisville, NC, USA). Brachial blood flow (BBF, ml/min) was assessed using a duplex ultrasound (LogiQ, GE Medical, Milwaukee, WI, USA) with an 8–12 MHz multifrequency linear transducer (11L) with a constant insonation angle of 60° relative to the skin. Arterial diameter was assessed using an automated system (Vascular Research Tools, Medical Imaging Applications LLC, Coralville, IA, USA) and blood velocity was obtained during the muscle relaxation phase of the dynamic HGE. Those values were averaged from the last 30 s of each protocol phase and brachial vascular conductance (BVC, ml/min/mmHg) was calculated as (BVC = BBF/MAP).

### 2.3 Experimental protocol

The experimental protocol was performed during one visit to the laboratory 2 h after a light breakfast. Upon arrival, the weight and height of the participants were measured. After that, participants were placed in a recumbent position and instrumented with a venous catheter in the antecubital vein, an electrocardiogram, a cuff on the left arm for brachial BP measurements and a cuff on the middle finger of the left hand for finger BP measurements. Later, the maximal voluntary contraction (MVC, N) was measured with an electronic handgrip dynamometer (PowerLab, LabChart; ADInstruments). Afterwards, participants sat quietly for at least 10 min until the variables were at baseline. Subsequently, participants underwent 3 min of rest, and 3 min of dynamic HGE at 50% of MVC (Control: 17.3 ± 4.4 kg, and post‐COVID: 15.4 ± 4.9 kg; *P* = 0.32). After the first minute of the HGE a bolus of 100 μg of SNP was systemically administered intravenously, followed by a bolus of 150 μg of PHE–HCl 1 min later in the non‐exercising arm. The dynamic exercise was performed with a metronome as auditory feedback at a frequency of 80 beats per minute (i.e., one beat for muscle contraction and two beats for muscle relaxation) and with visual feedback at the aimed force. All participants were right‐hand dominant. A scale from 1 to 10 was used to obtain the rating of perceived effort during the HGE (Borg, 1990).

### 2.4 Data and statistical analysis

Data are presented as means ± standard deviation and Δ values were calculated from baseline resting values. The data distribution was tested by the Shapiro–Wilk test, and all variables were normally distributed. Student's *t*‐test was applied to test possible differences between participants’ characteristics. The main effects of time during the HGE (control, SNP and PHE), group (Healthy Controls vs. post‐COVID) and their interactions were tested by applying a two‐way ANOVA with fixed effects (within–between). When significance was found for the interaction, the Bonferroni *post hoc* adjustment was used for multiple comparisons. Statistical significance was set at *P* ≤ 0.05, while an α‐value of 0.05 was assumed. Although the sample size was small, the possible sex differences were investigated, but no statistical differences were found. All analyses were performed using SPSS Statistics V20.0 software (IBM Corp., Armonk, NY, USA) and graphs were created using the GraphPad Prism 8 software (GraphPad Software, San Diego, CA, USA).

## 3 RESULTS

**FIGURE 1:**
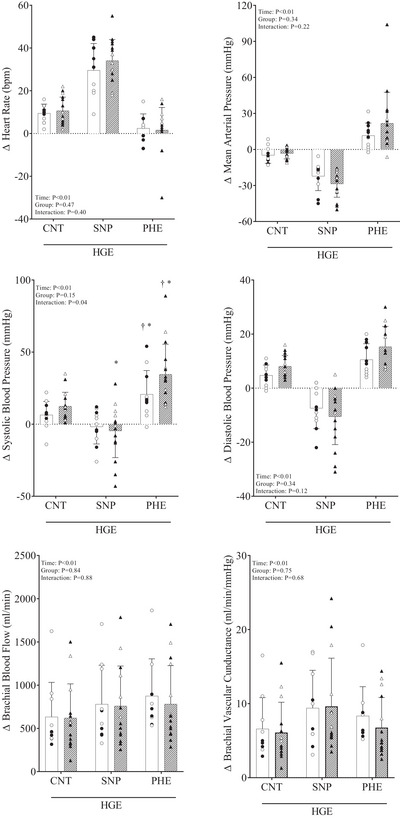
Cardiovascular response during dynamic handgrip exercise (HGE), with a control phase (CNT), a bolus of sodium nitroprusside (SNP) and phenylephrine hydrochloride (PHE). White bars with circles represent the control group (men *n* = 6 and women *n* = 5) and hatched bars with triangles represent post‐COVID (men *n* = 6 and women *n* = 10). Filled symbols represent women and open symbols represent men. Means ± SD, two‐way ANOVA with significance set to *P* ≤ 0.05. *P ≤ 0.05 vs. CNT and †*P *≤ 0.05 vs. SNP.

Participants' characteristics are shown in Table 1; the groups were not different in age, body mass index and maximal voluntary contraction. Meanwhile, resting HR, brachial BP, BBF and conductance were not different between groups (Table 2). Dynamic HGE increased HR and BP, in the control and post‐COVID groups. While the bolus of SNP evoked a reflex increase in HR in response to the decrease in BP, the bolus of PHE increased BP causing a reflex decrease in HR. No differences were observed between the control and post‐COVID groups in HR and BP (*P *> 0.05 between groups, Figure 1). BBF and conductance increased during the dynamic HGE, but SNP and PHE did not change the effect of HGE in the control group and post‐COVID group (Figure 1). Perceived effort during HGE in arbitrary units was not different between groups (Control 3.7 ± 2.8 vs. post‐COVID 4.0 ± 2.2; *P* = 0.73), and all participants reached the aimed force during the 3 min of exercise.

**TABLE 1:** Participant characteristics.

	Healthy controls	Post‐COVID‐19	*P*
Age (years)	28 ± 7	30 ± 9	0.37
Weight (kg)	77 ± 17	64 ± 10	0.03
Height (cm)	173 ± 10	166 ± 9	0.05
BMI (kg/m^2^)	26 ± 3	24 ± 3	0.07
MVC (kg)	35 ± 9	31 ± 10	0.18

*Note*: Healthy controls (*n* = 6 men and *n* = 5 women) and post‐COVID‐19 (*n* = 6 men and *n* = 10 women). Data are means ± SD, with *t*‐test with significance set to *P *< 0.05. BMI, body mass index; MVC, maximal voluntary contraction.

**TABLE 2:** Cardiovascular variables at rest.

	Healthy controls	Post‐COVID‐19	*P*
Heart rate (bpm)	65 ± 11	68 ± 11	0.46
Brachial systolic blood pressure (mmHg)	123 ± 8	123 ± 9	0.86
Brachial diastolic blood pressure (mmHg)	78 ± 8	77 ± 8	0.08
Brachial mean arterial pressure (mmHg)	93 ± 7	92 ± 8	0.82
Brachial blood flow (ml/min)	78.5 ± 46	70.3 ± 29	0.64
Brachial vascular conductance (ml/min/mmHg)	0.86 ± 0.52	0.78 ± 0.34	0.67

*Note*: Healthy controls (*n* = 6 men and *n* = 5 women), Post‐COVID‐19 (*n* = 6 men and *n* = 10 women). Data are means ± SD, with *t*‐test with significance set to *P *< 0.05.

## 4 DISCUSSION

The main findings of this study were that young healthy men and women who developed mild to moderate COVID‐19 present preserved vascular conductance at rest and exercise‐induced increase in skeletal muscle blood flow 12–14 weeks after the acute infection, contrary to our hypothesis. Additionally, our results indicate that young men and women who have recovered from COVID have preserved endothelial function as evidenced by preserved blood flow during HGE, and do not show an exaggerated vascular constriction in response to sympathetic stimulation.

Post‐infection symptoms and long COVID‐19 sequelae are still a matter of debate (Astin et al., 2023; Castanares‐Zapatero et al., 2022). Young patients 4 weeks after testing positive for COVID‐19 showed exaggerated BP and HR responses and decreased exercise‐induced hyperaemia during dynamic HGE at 30% and 45% of the maximal voluntary force (Stute et al., 2022). Additionally, brachial artery flow‐mediated dilatation is also impaired 3–4 weeks after COVID‐19 (Ratchford et al., 2021). Those responses could be due to an exaggerated sympathetic response or endothelial dysfunction. However, in our study, we did not observe impaired exercise‐induced hyperaemia during a HGE at 50% of maximal voluntary contraction. Therefore, in young healthy subjects, a possible impaired endothelial function caused by COVID‐19 ceases in a short time.

Exercise intolerance and fatigue seem to be common after recovery from acute COVID‐19 infection (Schwendinger et al., 2023; Weldon et al., 2023; Wernhart et al., 2023) and could be attributed to the interaction of several pathophysiological mechanisms in multiple organs and systems (i.e., ventilatory, cardiovascular, musculoskeletal), including vascular control of skeletal muscle blood flow during physical exercise. Therefore, impaired exercise‐induced hyperaemia could lead to decreased exercise capacity after COVID‐19 infection. However, in this study, the participants showed no signs of fatigue in either group; they reported that the exercise was moderate and somewhat hard (Borg, 1990) and reached the aimed force throughout the 3 min of the HGE.

Exercise‐induced hyperaemia is regulated to match metabolic demand through a balance between the vasoconstrictor effect of the systemic increase of sympathetic activity and the vasodilatory effect of the metabolites formed locally in the active skeletal muscle, including NO (Hellsten et al., 2012; Laughlin et al., 2012). In our study, an NO donor did not change the exercise‐induced hyperaemia in either group, suggesting that redundant mechanisms compensate to tightly regulate the flow according to metabolic demand. The NO donor did not change the exercise‐induced hyperaemia in the post‐COVID group, indicating that endothelial function might be preserved, contrary to our hypothesis.

A failure to control BP has been reported in patients after COVID‐19 infection (Elkholey et al., 2023; Ratchford et al., 2021), and increased vascular transduction and excessive vasoconstriction during sympathetic stimulation could explain that (Fairfax et al., 2013; Young et al., 2021). In this study, a bolus sympathetic agonist was infused intravenously during HGE. Nevertheless, BBF did not change in either group, demonstrating that vascular response seems to be preserved during sympathetic stimulation in young healthy subjects after recovery from COVID‐19.

### 4.1 Experimental considerations

The results of the present study must not be extrapolated to other populations, especially symptomatic patients, patients who presented severe COVID‐19 symptoms needing or not hospitalization, and patients who have any other chronic disease. Additionally, we must consider the possibility that participants in the control group had at any time point asymptomatic COVID‐19. The BP during exercise was measured using photoplethysmography on the finger, but it has been used in several protocols during exercise. The participants performed exercise using a small muscle mass, and the results could be different if the exercise were performed by a large muscle mass or during a high‐intensity whole‐body exercise. No statistical significance was found for sex differences. However, the small sample size must be considered for interpreting those results, and future research addressing the issue is needed. The methods used in this study did not allow the synchronized measurement of beat‐to‐beat blood flow and BP changes. However, the average of the 30 s period is adequate to respond to our research question.

### 4.2 Conclusion

The present study provides novel data supporting the conclusion that exercise‐induced hyperaemia is preserved in healthy young subjects 12–14 weeks after the recovery from mild to moderate symptomatic COVID‐19.

## AUTHOR CONTRIBUTIONS

Eliza Prodel Antonio C. L. Nobrega and Natalia G. Rocha contributed to the study's conception and design. Data analysis was performed by Eliza Prodel and Beatriz Divino Material preparation and data collection were performed by Eliza Prodel Roberto Souza, Helena N. M. Rocha and Beatriz Divino. The first draft of the manuscript was written by E.P. and all authors commented on previous versions of the manuscript. All authors have read and approved the final version of this manuscript and agree to be accountable for all aspects of the work in ensuring that questions related to the accuracy or integrity of any part of the work are appropriately investigated and resolved. All persons designated as authors qualify for authorship, and all those who qualify for authorship are listed.

## CONFLICT OF INTEREST

The authors declare no conflicts of interest.

## Data Availability

The data that support the findings of this study are available from the corresponding author upon reasonable request.
